# Guidelines for leadership development of youth with physical disabilities through leisure education: A Delphi study

**DOI:** 10.4102/ajod.v11i0.1073

**Published:** 2022-11-04

**Authors:** Makhaya J. Malema, Luzaan Africa, Linda Caldwell, Marie Young, Lisa Wegner

**Affiliations:** 1 Department of Sports, Recreation and Exercise Science, Faculty of Community and Health Sciences, University of the Western Cape, Bellville, South Africa; 2 Interprofessional Education Unit, Faculty of Community and Health Sciences, University of the Western Cape, Bellville, South Africa; 3 Department of Recreation, Park and Tourism Management, The Pennsylvania State University, Pennsylvania, United States; 4 Department of Occupational Therapy, Faculty of Community and Health Sciences, University of the Western Cape, Bellville, South Africa

**Keywords:** leisure education, leadership development, leadership skills, youth development, youth with physical disabilities

## Abstract

**Background:**

Youth with disabilities benefit by developing a skill set to help resolve any issues during their daily activities, including pursuits that lead to productive livelihoods. Acquiring leadership skills through leisure education programmes may be particularly effective for youth with disabilities to gain confidence in their leadership abilities.

**Objectives:**

This study aimed to develop and reach a convergence of opinions on the preferred elements of a leisure education programme to promote leadership development among youth with physical disabilities.

**Method:**

In this study, a three-round Delphi methodology was used. In the first round, 16 experts participated; in the second round, 14 experts participated; in the third round, nine participated. The first round of the Delphi method consisted of a qualitative questionnaire with open-ended questions, which assisted in developing guideline statements. The results from the first round informed the second and third rounds of the study. The guidelines were reviewed for consensus in subsequent rounds using a Likert scale format.

**Results:**

In the final round (third round) of the Delphi method, the expert panel consisting of nine participants agreed that leadership development for youth with physical disabilities could be promoted by leisure, recreation, sports and activities of daily living.

**Conclusion:**

These guidelines are essential in building resilience, empowerment and independence and can be seen as a positive contribution to communities with disabilities and young people with and without disabilities.

**Contribution:**

These guidelines would build capacity and resilience among youth and equip them with the skills and abilities to initiate leisure programmes.

## Introduction

Youth leaders can become change agents within their societies by developing skills to resolve issues that may arise during daily activities (Grenwelge, Zhang & Landmark 2010). It is argued that for youth with disabilities to attain collective independence from institutions and service providers, they must be able to influence each other within their community and nominate a leader (Dowse 2001). According to the National Consortium on Leadership and Disability for Youth (n.d.), young people must persuade their peers to lead themselves.

By building their capacity as leaders, youth would be able to recognise areas that need change and bring about needed changes (National Consortium on Leadership and Disability for Youth n.d.). Youth development is essential and can be championed by developing leadership skills to help youth with disabilities deal with daily challenges. Youth development programmes have the potential to develop and build the capacity for young people to face current and future obstacles through structured activities that offer learning opportunities (National Consortium on Leadership and Disability for Youth n.d.).

Youth with physical disabilities face daily challenges and often have no support to navigate societal barriers. One area of support that has received some attention is developing leadership skills among youth with physical disabilities. Angima, Etuk and Maddy (2016) caution, however, that just providing adequate resources (which can include equipment, support and training) is inadequate to foster leadership skills; therefore, this paper focuses on identifying elements of a leadership development programme that have the potential to provide effective leadership training for youth with disabilities.

The National Longitudinal Transition Study-2 (2004:2) in the United States of America estimates that less than 3% of youth with disabilities take part in youth development and leadership development opportunities. In the South African context, Van Niekerk (2014) reports that youth programmes organised through governmental structures are implemented with political agendas to mobilise youth into the political atmosphere while neglecting their skills development.

The current study forms part of a bigger project which argues that leisure education programmes can be used to promote leadership development. Historically, leisure education programmes have focused on opportunities designed to promote social skills among youth with disabilities (Cory 2004), quality of life, community development (Levy 2000), perceptions of leisure (Ertuzun 2015), adaptive coping skills, well-being (Hartman, Evans & Anderson 2017) and inclusive leisure service (Dattilo 2018). Furthermore, leisure education programmes have been used to support caregivers of people living with dementia (Carbonneau, Caron & Desrosiers 2011). There is an existing gap in the literature on leisure education as a tool for developing leadership.

## Literature review

Leadership skills are personal skills in which individuals build their capacity through various experiences and engagements. According to Fredriksson, Geidne and Eriksson (2018), developing personal skills involves offering support to an individual on a personal and social level and encouraging information sharing, training and education and lifelong skills. The concept of leadership is perceived and argued in the context that youth with physical disabilities can influence their peers, leading them during leisure education programmes. Leadership in this context involves an individual’s ability to influence a group of people to reach a shared objective (Redmond & Dolan 2014). Leadership requires an individual to lead and guide and people to follow.

It is proposed that through the guidelines developed in this study, youth with physical disabilities will influence their peers to be part of leisure education programmes as they grow into their leadership roles. In this study, knowledge about leisure education is seen as a mechanism to facilitate leadership development among youth with physical disabilities. Youth with physical disabilities need to know who they are leading and what goal is being achieved. Understanding the followers and goals will allow youth with physical disabilities as leaders to gain valuable, insightful and meaningful experiences from leisure education programmes to lead their peers successfully.

Leadership is a lifelong skill used daily among peers in a society and community. Being a leader of a minority group can be seen as an inspiration for other young people and may positively influence leisure participation. Influential leaders occupy multiple roles, including good communicators, enablers, innovators, teachers, coordinators, motivators, problem solvers and decision-makers (Russell 2001). Participants are presented with an opportunity for self-expression by promoting creativity, fantasy and progression toward personal potential; learning and growth are presented by opportunities for learning and growth, exploration and exposure to new facts and ideas (Edginton & Edginton 1994).

### The role of leisure education

Leisure education can facilitate skills development and build capacity among youth with physical disabilities. Leisure is valued for offering opportunities for youth with disabilities to express, explore, discover, create, exchange and communicate meaningfully with their peers (Edginton 2006). According to Segve (2018), a leisure education programme has three purposes: (1) embracing the outdoor environment and activities, sports and diversity of games and play; (2) facilitating fun and enjoyable activities such as cooking, gardening, watching television and participating in leisure-time physical activities; and (3) including activities that are interpersonal-social. Thus, it is important for youth with physical disabilities to engage in leisure education programmes to develop valuable skills while benefiting from fun and enjoyable activities.

The Charter for Leisure Education and Recreation Association (1993) and Sivan (1997) stated that leisure education refers to a focused, methodological and well-aligned process that recognises an individual’s choice and right to leisure, and the meaningful use of it, to influence and enable desirable patterns of leisure behaviour. In this study, leisure education is adopted as the lifelong learning process that facilitates leisure-related skills and positive values and attitudes by using leisure activities to develop themselves within a leisure context (Dieser 2012; Sivan 1997; Stebbins 1999). This definition is aligned with the charter’s objective about leisure education. It aims to inform and guide stakeholders such as governments, nongovernmental organisations and educational institutions about the importance and benefits of leisure and education (Sivan 1997). The current study argues that youth with physical disabilities can use leisure education programmes as a tool to develop and nurture their leadership skills. It is acknowledged that youth with physical disabilities would need to collaborate and actively contribute to leisure education programmes within their communities.

### Leisure education as means to develop leadership

According to the Swedish National Agency for Education (2014), good quality leisure-time centres depend on competent teachers and pedagogues who can lead and implement the programmes and activities according to the curriculum and published research. Thus, leisure services and programme providers have an important role in developing, transferring and nurturing skills and knowledge for the benefit of the participants. Bengu (former Minister of Education in South Africa) called for education and training change (Department of Education and Training [DET], Parliament of South Africa 1995). He stressed the importance of a:

[*N*]ational project of reconstruction and development which compels everyone in education and training to accept the challenge of creating a system that cultivates and liberates all people’s talents without exception. (p. II)

The current study embraces the same notion by the South African Department of Education and the Swedish National Agency for Education. Youth with physical disabilities can thus develop leadership skills through leisure education activity programmes. The Swedish National Agency for Education (2014:14) suggested that learning programmes in leisure-time centres could be formal, informal and flexible to create accommodating and stimulating environments where the interests of the participants are at the forefront of planning. The current study is underpinned by leisure education and leadership development principles. The elements within each domain offer an opportunity for youth with physical disabilities to be developed as leaders using leisure education as a tool.

### How leisure education can develop leadership skills

Leisure education programmes can help develop and promote lifelong skills such as leadership abilities for participants, regardless of physical, intellectual and other limitations (Segve 2018). According to Malema, Young and Wegner (2022), the ability of youth with physical disabilities to develop leadership rests on their active engagement in leisure education programmes. Additionally, youth with physical disabilities can advance their self-development through opportunities that allow them to explore and identify their leisure time needs, thereby building their capacities as leaders. The authors further argue that leadership development shows that leisure education is appropriate for youth with disabilities to become leaders.

Furthermore, Jooste (2019) argued that building networks and relationships are crucial for youth with physical disabilities to develop as leaders. This enables youth to seek support and build up their capacity in areas they lack, allowing them to be the leaders their peers can follow. Leadership capacity can be facilitated through developing and practising leadership skills that match youth’s personal abilities (Sivan 2014, 2017; Sivan & Chan 2012). Therefore, leisure education programmes must facilitate and provide opportunities for youth with disabilities to demonstrate an application of their leadership skills (Jooste 2019). Additionally, because decision-making is an important component of leadership, youth with physical disabilities must be able to demonstrate and develop their abilities to make decisions during their leisure engagement (Sivan 2014, 2017; Sivan & Chan 2012).

Facilitating leadership development in leisure education programmes also implicates the community context. Leisure service providers are challenged and encouraged to offer opportunities for social activities that can influence the attitudes of community members towards positive participation and being mindful of the use of language that promotes people’s dignity and that advocates for the communities they live in (Dattilo 2018). Albertyn and Frick (2016) argue that leadership development efforts must focus on the skills relevant to the current diverse and challenging times. This article reports the results of a Delphi study conducted with an expert panel to develop a consensus around the preferred elements of a leisure education programme designed to promote leadership among youth with physical disabilities. Therefore, this study aims to develop and reach a consensus on the preferred elements of a leisure education programme to promote leadership development among youth with physical disabilities.

## Methodology

### Design

A three-round Delphi method was used to design and develop guidelines for youth with physical disabilities. Grobbelaar (2007) refers to the Delphi method as a research methodology exploring the anticipated future of innovative and evolutionary phenomena. Jünger et al. (2017) described the aim of the Delphi method as the formation of consensus and/or explanation of a topic beyond existing knowledge and the present conceptual world. This method is based on the premise that well-informed individuals, drawing on their perceptions and prior experience on the topic of study, are better prepared to estimate the future than theoretical approaches or trends (Grobbelaar 2007).

### Participants

The recruitment and selection of the experts in this study followed a standard protocol (Grime & Wright 2014). A panel of experts was identified using purposive sampling. The inclusion criteria were that participants could include academics, researchers, professionals, practitioners, programmers, service providers, people living with disabilities and activists who had the knowledge and/or expertise in leisure and recreation, youth and leadership development and youth or disability studies. A total of 37 eligible experts were identified and were sent an information letter via e-mail explaining the procedure of the Delphi method and expectations should they agree to participate. The experts were identified through their literature contribution to leisure and leisure education, and their research on people with disabilities. The same experts completed each round, excluding those who dropped out in round two.

In each round of the Delphi communication process, three e-mails were sent out to the participants who acknowledged and indicated their interest in the communications. The first e-mail was the official list of questions that required their engagements. This was followed up by two e-mail reminders at least 3 weeks apart to remind expert panel members who may not have engaged in the discussion. Participants were perceived to have dropped out when they failed to acknowledge the e-mail or confirm their interest in participating further in the study.

### Data collection

Data collection was done through three rounds. The research team used Google Forms (Alphabet Inc., Mountain View, California, United States) to distribute the self-administered questionnaire to the expert panel, and the Google Form automatically saved the participants’ responses into an Excel spreadsheet (Microsoft Corporation, Redmond, Washington, United States). The following eight themes were developed as a synthesis from the findings of the bigger project which include scoping review, quantitative and qualitative approaches: (1) feasible strategies for developing leadership skills in youth with physical disabilities; (2) feasible out-of-school approaches for leisure activity programmes that follow a nonformal structure; (3) guide to help youth with physical disabilities realise their maximum leadership potentials; (4) implementing leadership skills during leisure education programmes; (5) implications for allowing youth to take the lead in their leisure activities; (6) role modelling and peer support among youth in communities or societies; (7) balancing leisure education programmes to promote holistic development; and (8) the benefits of knowledge sharing for leadership development.

In this investigation, the first round of the Delphi study consisted of a qualitative questionnaire with open-ended questions to gather information from an expert panel. This first round explored the experts’ perceptions on guidelines for leadership development using leisure education as a tool. The expert panel was expected to comment on how those questions can develop youth into leaders during leisure education. The feedback from round one was used to develop the guidelines, which informed the subsequent rounds of the study. In the second round, the guidelines developed in round one were used to develop a Likert scale survey. The iterative process was continued until 70% consensus was reached, which indicates that theoretical saturation was achieved (Skulmoski, Hartman & Krahn 2007). In round three, the panel of experts validated round two as an accurate reflection of how the guidelines can facilitate leadership development using leisure education. Examples of the statements and questions are the following:

**Statement:** Activities that can promote leadership development include leisure, recreation, sports and physical activity programmes and activities of daily living.

**Question:** How can these activity programmes be implemented to develop leadership during leisure education programmes?

**Statement:** Leisure activity programmes in an out-of-school context are recommended. Such activities can use a nonformal structure, making learning and development specific as per the participant, for example, youth with a physical disability.

**Question:** How feasible is it to use an out-of-school approach to implement leisure education programmes for leadership development among youth with physical disabilities?

**Statement:** It is recommended that youth with physical disabilities identify their leadership skills and abilities that they can develop further during leisure activities.

**Question:** How can youth with physical disabilities realise their leadership skills?

### Data analysis

This study used thematic analysis to analyse and present data for round one. The researcher read the responses from each expert separately. Notes were made in the margins to highlight guidelines recommended for each of the eight themes. Using the notes and responses from this round, the researchers applied a deductive analysis approach to present the guidelines relevant to developing youth with physical disabilities to become leaders. The data from round two were analysed through a basic descriptive statistical analysis to obtain the percentage as a level of agreement from the expert panel using the IBM SPSS statistics version 27 (IBM Corporation, Armonk, New York, United States) (Goerge & Mallery 2019). For round two, the guidelines were rated using a five-point Likert scale with the following ratings: 1 = strongly disagree, 2 = disagree, 3 = not sure, 4 = agree and 5 = strongly agree. These ratings were used to determine consensus among the expert panel. The research team checked the completeness and correctness of the responses on the questionnaire items. The current study adopts the stance of Hsu and Sandford (2007) and Boulkedid et al. (2011). They recommend that at least 70% of expert participants rate three or higher on a five-point Likert type scale and that the median score must be greater than 3.25 to demonstrate consensus. Furthermore, the current study’s five-point Likert scale was grouped into three categories: nonconsensus (‘strongly disagree’ and ‘disagree’ ratings), consensus (‘strongly agree’, ‘agree’ ratings) and ‘neutral’.

### Ethical considerations

This study received ethical approval from the Biomedical Research Ethics Committee at the University of the Western Cape (ethical clearance number BM20/2/1). All participants gave informed consent and were informed about their right to withdraw from the study without repercussion.

## Results

The results of this study reports findings from data collected through qualitative and quantitative methodology which formed the current Delphi technique. The demographic information of this study is reported in Table 1.

**TABLE 1:** Demographic information of the participants in round one.

Participants’ location	Areas of expertise
**Round one demographic information**	
South African experts *n* = 9 (56.25%)	Leisure and recreation experts *n* = 9 (56.25%)
Turkey *n* = 2 (12.5%)	Disability activists *n* =3 (18.75%)
USA *n* = 2 (12.5%)	Youth and leadership developmental experts *n* = 3 (18.75%
Australia *n* = 1 (6.25%)	Occupational therapist *n* = 1 (6.25%)
Canada *n* = 1 (6.25%)	-
Malaysia *n* = 1 (6.25%)	-
**Round 2 demographic information**	
South Africa *n* = 9 (64.3%)	Leisure and recreation experts *n* = 7 (50%)
Turkey *n* = 1 (7.14%)	Disability activists *n* = 4 (28.57%)
USA *n* = 1 (7.14%)	Youth and leadership developmental experts *n* = 2 (14.29%)
Australia *n* = 1 (7.14%)	Occupational therapist *n* = 1 (7.14%)
Canada *n* = 1 (7.14%)	-
Malaysia *n* = 1 (7.14%)	-
**Round 3 demographic information**	
South Africa *n* = 9 (100%)	Leisure and recreation experts *n* = 4 (44.44%)
-	Disability activists *n* = 4 (44.44%)
-	Youth and leadership developmental expert *n* = 1 (11.12%)

In round one, 16 experts out of 37 (43% participation rate) agreed to be part of the Delphi study and completed the open-ended questions. These participants were geographically diverse and included national (South Africa–based) and international experts. In round two, 14 experts out of 16 (attrition rate of 12.5%) completed the Likert-scale questionnaire. In round three, nine participants out of 14 (attrition rate of 35.7%) from South Africa completed the questionnaire and confirmed the guidelines as a true reflection. Only South Africa–based experts completed round three. The expert panel in this round were purposively selected because of their perceived understanding and experiences of the South African context, culture and communities, being residents in the country.

The following forms part of the theme statement and guidelines formulated and agreed upon by the expert panel.

### Theme one: Feasible strategies for developing leadership skills in youth with physical disabilities

The expert panel reported that consideration of the context plays an essential role in leisure, recreation, sports, physical activities and activities of daily living to facilitate leadership development for youth with physical disabilities. Participants expressed that various factors need to be implemented to ensure leadership development. Tables 2–9 presents feasible strategies for developing leadership skills in youth with physical disabilities as shared by the panel of experts. Participants were asked to rate these guidelines in round two based on the guidelines identified in round one. In round two, participants agreed that all five guidelines could facilitate youth with physical disabilities to develop leadership during leisure education programmes.

**TABLE 2:** Guidelines for leadership development through leisure, recreation, sports, physical activities and activities of daily living.

Guideline statement	Consensus %
1. Specific outcomes that focus on leadership development	100
2. Youth should play an active role in the programme designs	100
3. Shared leadership	92.8
4. Mentorship, coaching and knowledge-sharing opportunities	100
5. Being sensitive towards people with disabilities and working towards eliminating barriers that hinder participation in societal activities	92.8

**TABLE 3:** Guidelines for an out-of-school approach to leisure education programmes.

Guideline statement	Consensus %
1. Elements of intrinsic motivation (relatedness, competence, autonomy) should be part of the programme for sustained participation	100
2. Freedom of choice principle for all participants and support for everyone to be engaged	92.8
3. Access and availability to resources, opportunities and equipment (including transportation, facilitators, coaches and human support and assistance) after hours	92.8
4. Soliciting and recognising input from youth on programmes offered	92.8

**TABLE 4:** Guidelines for youth with physical disabilities to realise their leadership skills.

Guideline statement	Consensus %
1. Elements of self-knowledge in the programmes	92.8
2. Youth must be able to choose their level of involvement and engagement in programmes	85.7
3. Giving youth the power to drive the process and own their leisure activities	100
4. Positive reinforcements by showing youth how to do the activity and offering support where needed	92.8
5. Partnering with NGOs, local sports, recreation and culture departments, municipalities and other relevant stakeholders	100
6. Exposure to situations that promote youth to exercise leadership skills	100

NGO, nongovernmental organisation.

**TABLE 5:** Guidelines on how leadership skills can be developed during leisure education programmes.

Guideline statement	Consensus %
1. Programmes should be based on reciprocal experiential learning	100
2. Allow youth to take ownership of the programme and offer them opportunities to apply their leadership skills during activities	100
3. Introduce the principles of reflection and progression practices	100
4. An atmosphere should be created for youth to realise their talents and other potential abilities	100
5. Leisure education programmes should include equity, diversity and inclusion as principles	92.8

**TABLE 6:** Guidelines of the implications for allowing youth to take the lead in facilitating their leisure activities.

Guideline statement	Consensus %
1. A good foundation and adequate support for youth is needed to allow freedom for them to facilitate their own experiences	100
2. Learning through practice and offering the necessary support	100
3. Offers youth an opportunity to improve through information sharing or interchange during leisure context	100
4. Youth must be made aware of the importance of decision-making, communication and behaviour as factors that can influence their success as leaders	92.8
5. Youth can develop sound ethics, self-confidence, happiness and peace and can benefit society and their immediate surroundings	85.7

**TABLE 7:** Guidelines on role modelling and peer support among youth with disabilities within their communities and societies.

Guideline statement	Consensus %
1. Youth should be offered opportunities to progress from merely participating in programmes to playing an active role in the programmes and gaining broader community involvement	100
2. Encouraging reflective practices will reinforce the leadership role and allow leadership skills to be applied in other settings	100
3. Mentorship and peer influence among youth with physical disabilities can promote their empowerment in society	100
4. Positive reinforcement can be used to strengthen their leadership qualities, boost their morale and motivate them	100
5. Encourage youth to be engaged in communities to promote self-trust, develop skills and diversify their social activities	100

**TABLE 8:** Guidelines on balancing leisure education programmes to promote holistic development among youth with physical disabilities.

Guideline statement	Consensus %
1. Using intentional programming and a logic model for programme planning	92.8
2. Offering adequate support for youth and exposing them to real-life scenarios	100
3. Incorporating youth development principles to ensure a holistic approach and avoid a singular focus	100
4. Offer programmes that allow a variety of skills that are guided and ensure that youth have an input and a voice	100
5. Centre the programmes around individuals’ needs and consider individuals’ abilities, capabilities and strengths	100

**TABLE 9:** The benefits of knowledge sharing for leadership development among youth with physical disabilities.

Guideline statement	Consensus %
1. Sharing knowledge can assist youth in identifying their strengths and weaknesses and also create an interest in leadership	100
2. Youth with disabilities can be nurtured to understand their potential and how new knowledge will assist them in growing and applying the new knowledge in settings beyond the leisure experience	100
3. Leisure education programmes build youth’s capacity, empowerment and application of the skills they learn	100
4. Knowing what works and what does not. Understanding what resources are needed and then doing what works for persons with disabilities in the programme	100
5. The more accurate the information people have, the more they know their strengths and weaknesses and try to correct their deficiencies and improve on their shortfalls	100
6. Youth can be equipped to take the initiative and have self-reliance, self-development and awareness	100
7. Delivering knowledge and improving awareness of the issue is a good step in providing youth with more leisure opportunities	100

### Theme two: Feasible out-of-school approach for leisure activity programmes that follow a non-formal structure

The expert panel shared their perceptions on how an out-of-school approach needs to consider elements that facilitate independence, support and a less restrictive environment. In round two, participants agreed that all five guidelines are feasible during an out-of-school approach for a leisure education programme.

### Theme three: Guide to help youth with physical disabilities realise their maximum leadership potential

Participants recognised the importance of youth with physical disabilities realising their leadership potential to facilitate their development. In round two, they agreed that all six guidelines are important to consider, and careful attention is required for youth with physical disabilities to be developed as leaders.

### Theme four: Implementing leadership skills during leisure education programmes

Participants shared their perceptions on what they considered feasible for leadership development using leisure education programmes for youth with physical disabilities. Participants were asked to rate these guidelines in round two based on the guidelines identified in round one. In round two, participants agreed that all five guidelines could guide youth with physical disabilities to be developed as leaders using leisure education as a tool. Careful planning and individual consideration are needed to successfully develop youth as leaders.

### Theme five: Implications for allowing youth to take the lead in their leisure activities

The expert panel shared their knowledge of the implications of allowing youth with physical disabilities to take the lead in facilitating their leisure activities. In round two, participants agreed that all five guidelines have good implications and promote youth with physical disabilities to take the lead in facilitating their leisure activities. The proposed guidelines allow youth to learn, grow and develop through active engagement when taking the lead in their preferred leisure activities.

### Theme six: Role modelling and peer support among youth in communities or societies

The expert panel shared their perceptions on how youth with physical disabilities can be developed as leaders by embracing role modelling and offering peer support within their communities and societies. In round two, participants agreed that all five guidelines help youth with physical disabilities become role models and offer peer support within their communities and societies.

### Theme seven: Balancing leisure education programmes to promote holistic development

The expert panel agreed that balanced leisure education programmes could facilitate holistic development among youth with physical disabilities. In round two, participants agreed that all five guidelines are essential when planning a balanced leisure education programme to promote holistic development among youth with physical disabilities.

### Theme eight: The benefits of knowledge sharing for leadership development

Participants reported on their perceptions of knowledge-sharing for leadership development among youth with physical disabilities. In round two, participants agreed that all seven guidelines are essential knowledge-sharing elements to develop leadership in youth with physical disabilities.

## Discussion

This study established guidelines that enable youth with physical disabilities to develop leadership skills using leisure education programmes. Participants agreed that leadership development for youth with physical disabilities could be promoted by leisure, recreation, sports and activities of daily living. These guidelines will assist youth in developing the capacity to take the initiative and plan their leisure programmes. According to Olsen and Burk (2017), the challenge of developing leadership is not teaching leadership concepts but instead developing leadership skills through a practical application of strategies and guidelines. Leisure, recreation, sports, physical activities and activities of daily living offer practical exposure to youth with physical disabilities. Schreuer, Sachs and Rosenblum (2014) also argued that involvement in discretionary play and leisure activities is significant for developing essential skills, self-identity and well-being. Therefore, the present study’s guidelines are feasible to follow and have an impact on youth, which suggests that they can be implemented successfully.

Additionally, the findings from the present study recommend that an out-of-school context would enable leadership development among youth with physical disabilities. The idea of an out-of-school context is ideal based on the notion that youth with physical disabilities should rely not only on formal school or institutional programmes but also programmes within their communities where they can decide when and how to be part of the programme. Dowse (2001) noted that youth with disabilities should collectively participate in political activities to influence policies and how they are implemented in their local communities, with an overall target to facilitate social transformation. This perception can contribute to youth taking ownership of their leisure spaces.

Furthermore, the findings of this study suggest that youth with physical disabilities can be supported to realise their leadership skills. Jacobsen and Thorsvik (2008) proposed that leadership can be seen as a series of actions by one or more people and that leadership is focused on facilitated learning. Augustsson (2018) stated that leadership is appropriate when others need to be influenced or persuaded. Therefore, this study argues that a perception that youth with physical disabilities can influence and persuade their peers during leisure education programmes leads to them becoming leaders.

In the current study, participants reached a consensus on guidelines of how leadership skills can be developed during leisure education programmes. The guidelines reported in this study illustrate that youth with physical disabilities can be held accountable and responsible for their leadership development. Leisure service providers could be supportive agents of the guidelines from this study. Various programmes can enhance this approach for youth that are more than merely fun activities (Caldwell 2000). Considering the statement by Caldwell (2000), when youth are engaged in a leisure education programme to develop and build their skills and promote their abilities, leadership becomes inevitable.

The current study recommends allowing youth to take the lead in facilitating their leisure activities. These guidelines constitute strategies that enable youth to be at the forefront of their leisure education programmes, allowing them to exercise their leadership development. This notion calls for consultation and needs assessment when planning leisure and recreation programmes explicitly involving youth with physical disabilities. Wilson (2000) shares that for too long, participants accepted whatever programmes had been planned and implemented for them.

The expert panel embraced the guidelines on role modelling and peer support among youth with physical disabilities within their communities and societies. According to Allman and Cutforth (2014), successful leadership within the sports sector depends on the ability to unite under a feasible vision and secure stakeholders to deliver on it. Therefore, it is essential for youth with physical disabilities to be united with mainstream society and not operate in isolation. Kim et al. (2016) suggest that people with disabilities use leisure to promote life satisfaction, foster social relationships, enhance self-esteem and confidence and cultivate hopefulness and happiness. Wilson (2000) recommends a need for new approaches to recreation planning, programming and delivery of services to accommodate the interest and needs of all population groups. This statement includes youth with physical disabilities who form part of society.

The findings of this study reported consensus on guidelines about balancing leisure education programmes to promote holistic development among youth with physical disabilities. Elements of these guidelines advocate for a supportive environment which enables holistic development among youth with physical disabilities. Dryfoos (1998) suggests that leisure and recreation programmes offer youth the relevant and necessary skills to overcome daily challenges and build resilience. Similarly, Green, Kleiber and Tarrant (2000) argue that intervention programmes can challenge youth mentally, socially and physically and provide the opportunity to facilitate long-term change and growth. Therefore, it is anticipated that by following these guidelines, the youth can become leaders for themselves and their peers and be equipped to deal with daily challenges.

Furthermore, the expert panel in this study acknowledged the benefits of sharing knowledge to promote leadership development. The guideline elements suggest that youth with physical disabilities must own and have access to information and have adequate support to develop their leadership. Albertyn and Frick (2016) recommend exploring change and knowledge management in a collaborative process, assisting in implementing strategies for progress in education. Although Albertyn and Frick’s study focuses on education within higher education, a case is made that the same sentiments can apply to leisure education programmes. Therefore, it is anticipated that the present study’s guidelines can be used successfully by youth with physical disabilities. Shinew, Hibbler and Anderson (2000) suggest that the youth must be educated about the relevant skills to navigate their daily challenges. These guidelines are essential in building resilience, empowerment and independence and can be seen as a positive contribution to communities with disabilities and young people with and without disabilities.

## Conclusion and future studies

The guidelines developed in the present study can be used to implement and initiate leisure activity planning, develop individual capacities and nurture leadership skills among youth with physical disabilities. The guidelines aim to equip and allow youth with physical disabilities to develop as leaders within their communities and peers, take the initiative and plan their leisure programmes. The guidelines in this study can be considered feasible and beneficial to youth with physical disabilities. For these guidelines to be fully implemented, youth with physical disabilities must be actively involved in leisure education programmes to develop leadership skills. It is concluded that leadership skills acquired and developed through leisure education programmes can be used outside the programmes to navigate day-to-day and life situations. Therefore, the researchers recommend that future studies investigate the influence of implementing the guidelines on youth leadership development.

## Strengths and limitations of the study

The guidelines formulated in this study are realistic and can bridge the exclusion gap for people with disabilities in South Africa. The guidelines can be adopted in other parts of the world to evaluate their impact within different settings. The significance and contribution of these guidelines lay a good foundation for more research within the disability communities. The limitations of this study include restricted expert panel availability, low response and a high dropout rate. Despite the study’s limitations, the positive contribution of this study outweighs the limitations.
